# 

**Published:** 1899-04

**Authors:** 


TABLE OF CONTENTS ON LAST ADVERTISING PAGE.

VOL. XIV. APRIL, 1899. No. 10.

### TEXAS Medical Journal.

SUBSCRIPTION, $1.00 A YEAR, IN ADVANCE.

SINGLE COPIES, 10 CENTS.

LIBRARY
SURGEON GENERAL'S OFFICE
APR.-12.-1899

PUBLISHED AT AUSTIN, TEXAS, BY

F. E. DANIEL, M. D., & S. E. HUDSON, M. D.,

EDITORS AND PROPRIETORS.

Eastern Representative: John Guy Monihan, St. Paul Building, 220 Broadway, New York City.

Entered at the Postoffice at Austin, Texas, as Second-class Mail Matter.

